# Classification of emotional states from electrocardiogram signals: a non-linear approach based on hurst

**DOI:** 10.1186/1475-925X-12-44

**Published:** 2013-05-16

**Authors:** Jerritta Selvaraj, Murugappan Murugappan, Khairunizam Wan, Sazali Yaacob

**Affiliations:** 1 Intelligent Signal Processing Research Cluster, School of Mechatronic Engineering, Universiti Malaysia Perlis (UniMAP), Kampus Ulu Pauh, Arau, Perlis, 02600, Malaysia

## Abstract

**Background:**

Identifying the emotional state is helpful in applications involving patients with autism and other intellectual disabilities; computer-based training, human computer interaction etc. Electrocardiogram (ECG) signals, being an activity of the autonomous nervous system (ANS), reflect the underlying true emotional state of a person. However, the performance of various methods developed so far lacks accuracy, and more robust methods need to be developed to identify the emotional pattern associated with ECG signals.

**Methods:**

Emotional ECG data was obtained from sixty participants by inducing the six basic emotional states (happiness, sadness, fear, disgust, surprise and neutral) using audio-visual stimuli. The non-linear feature ‘Hurst’ was computed using Rescaled Range Statistics (RRS) and Finite Variance Scaling (FVS) methods. New Hurst features were proposed by combining the existing RRS and FVS methods with Higher Order Statistics (HOS). The features were then classified using four classifiers – Bayesian Classifier, Regression Tree, K- nearest neighbor and Fuzzy K-nearest neighbor. Seventy percent of the features were used for training and thirty percent for testing the algorithm.

**Results:**

Analysis of Variance (ANOVA) conveyed that Hurst and the proposed features were statistically significant (p < 0.001). Hurst computed using RRS and FVS methods showed similar classification accuracy. The features obtained by combining FVS and HOS performed better with a maximum accuracy of 92.87% and 76.45% for classifying the six emotional states using random and subject independent validation respectively.

**Conclusions:**

The results indicate that the combination of non-linear analysis and HOS tend to capture the finer emotional changes that can be seen in healthy ECG data. This work can be further fine tuned to develop a real time system.

## Background

The expression and understanding of emotions play a major role in human communication facilitating mutual sympathy [1,2]. Extending this to machines and computers is one of the important issues that researchers in human-computer interaction (HCI) are trying to address [2]. Providing such sympathy would help humans to interact naturally with computers or machines improving the quality of service offered [3]. Though machines may never need all the emotional skills of people, equipping them with some of the skills will make them appear intelligent when interacting with people [4]. Such a system which can understand human emotions is also helpful in medical applications for treating patients with intellectual disabilities and autism [5,6].

**Figure 1:**
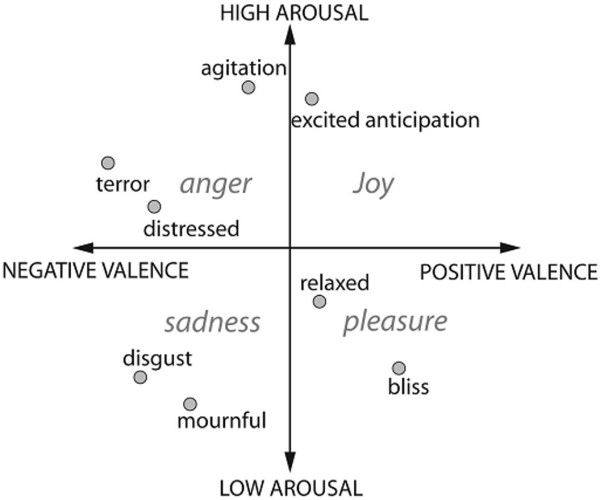
**Dimensional model of emotions **[2]**.** This model defines emotions on a two dimensional space – valance and arousal. Valance denotes the polarity of emotions (positive or negative) and arousal denotes the intensity (high or low). All the emotions can be plotted on this valance-arousal plot.

Emotions can be defined as a mental state that occurs spontaneously without any conscious effort and is accompanied by physiological changes. It is systematically produced by cognitive process, subjective feelings, physiological arousal, motivational tendencies, and behavioral reactions [2]. They are believed to interact with the mathematical, verbal and perceptual intelligence associated with the human brain [4]. Researchers have widely focused on two models of emotions – discrete and dimensional. The discrete model includes six basic emotions (happiness, sadness, fear, surprise, disgust, anger) that are universally accepted. All other emotions are considered to be a part of these basic emotions [7]. The dimensional model, as in Figure 1, plots emotions on two scales – valance and arousal. Valance denotes the polarity of emotion and arousal denotes the intensity of emotion. All emotions can be plotted on the valance-arousal plot [8]. Researchers have also proposed a three dimensional model of emotions which takes into account the attention-rejection property in addition to the two-dimensional model [2].

The approaches used for automatic emotion recognition, focus mainly on the audiovisual channels of emotion expression such as facial expression, speech and gestures [2]. Though these modalities are researched widely and have produced higher results, they are all prone to social masking. Emotions that are not expressed, emotions expressed differently (an angry person may smile) or minor emotional changes that are invisible to the natural eye cannot be tracked by these modalities [2,9-11]. Physiological signals being an activity of the autonomous nervous system (ANS) overcome these limitations as they reflect the inherent emotional state of the person. However, not much work has been done because of its complex nature and inability to visually perceive the emotion from the signal [1]. Physiological signal based emotion recognition also provides an opportunity to track minute emotional changes which cannot be perceived visually or by hearing [12].

Physiological measures such as Electroencephalogram (EEG), Electrocardiogram (ECG), Electromyogram (EMG), Galvanic Skin Response (GSR), Blood Volume Pressure (BVP), and Skin Temperature (ST) have been used to evaluate the emotional states of a person. QRS complex, an activity of the ANS that is derived from ECG provides information of the myocardial conduction system and can be used to understand the emotions experienced by a person [13]. This research being in the infancy stages, the performance of the different systems developed to recognize human emotions varies from 37% to 100%, depending on factors such as the number of emotions, the number of subjects, type of processing and types of emotion elicitation [2,3,14,15]. Some of the works are done in a subject dependent way where the training and testing data belong to the same subject [4,15].

Researchers have been using non-linear analysis in diverse areas of bio-signal processing for evaluating heart rate, renal activity, renal blood flow, arterial pressure, muscle activity and stress using signals such as Electrocardiogram (ECG), Heart Rate Variability (HRV), Electroencephalogram (EEG), Electromyogram (EMG) and Respiration Rate [16-18]. Non-linear analysis based on chaos theory helps in identifying the apparently irregular behaviors that are present in the system [19]. Non-linear features such as approximate entropy (APEN), largest Lyapunov exponent (LLE), correlation dimension (CD), Hurst exponent (H) and non-linear prediction error has been learned widely [16,20]. These features convey information related to properties such as similarity, predictability, reliability and sensitivity of the signal.

Hurst exponent analyzes the smoothness of a time series and is based on self similarity and correlation properties. It also evaluates the presence or absence of long-range dependence and its degree on a time series [16,21]. Emotions being transient the momentary variations that occur in the physiological signals can be evaluated by understanding the degree of similarity and short term correlations that can be measured using the Hurst parameter. Different methodologies based on rescaled range statistics, finite variance scaling (FVS), wavelet transform (WT) and empirical mode decomposition (EMD) are used to obtain the value of Hurst [20,22]. Previously, Hurst analysis has been done on various Biosignals for applications involving premature heart beat detection, coronary heart disease, sleep apnea and identification of mental state using EEG, HRV and ECG signals. Tommaso Costa et al., has examined Hurst exponent for cardiac signals in response to positive and negative stimuli [23]. However, not much work has been done to find the emotional content in the Hurst parameter.

Higher Order Statistics (HOS) refers to functions of orders three or more in contrast to the conventional statistics which are of orders one and two. The non-linear and non-Gaussian characteristics of the signal can be obtained by using HOS features and are widely used in the analysis of physiological signals [24,25]. Recently in [26], researchers achieved an 82% accuracy in recognizing emotions from EEG signals using HOS [26]. Their works indicate that HOS can also be used to seek emotional information from other physiological signals.

HOS features namely skewness and kurtosis measure the presence of transients in the signal and are robust to noise. Hurst measures the degree of self-similarity in the time series. In [27], the authors merged HOS with wavelets in order to compress ECG data and proved the merged method to efficiently exploit the inter beat correlations of the ECG data in noisy environments. In a similar way merging HOS with Hurst would enhance the variations in transient, thereby helping to effectively measure the short term correlations or the sudden physiological changes that occur during emotions.

In this work, we try to (1) understand the emotional information in ECG signals for the six basic and universal emotional states worked by Ekman et al., (happiness, sadness, fear, surprise, disgust and neutral) and (2) identify new non-linear features combining HOS and Hurst that can capture better emotional information from the physiological data [7]. Hurst and the proposed features were extracted from the QRS complex of emotional ECG signals using two widely used methods - Rescaled Range Statistics (RRS) and Finite Variance Scaling (FVS). The methodology, including emotion induction and data acquisition for the six emotional states (happiness, disgust, sadness, fear, surprise and neutral) is discussed in detail. Two new Hurst features based Higher Order Statistics (HOS) such as skewness based hurst and kurtosis based hurst are derived. These features were found to contain better emotional information compared to the Hurst derived in the traditional way. The performance, advantages and pitfalls are also discussed.

## Methods

### Emotional data acquisition

Gathering good and meaningful data is necessary in any signal processing application. In works related to emotion recognition using physiological signals, acquiring emotional physiological data is challenging because of the subjective nature of emotions and cognitive dependence of physiological signals. This necessitates the six emotional states to be elicited internally in the subject unlike other modalities of facial action or speech where the emotions can be enacted. The intensity of emotion induced varies among subjects and depends on psychological factors such as attention, orientation, social interaction and appraisal [2,4].

Researchers have used different methods to elicit the target emotions. Visual based elicitation using images, audio based elicitation using music and audio-visual elicitation using short film video clips are commonly used [2,15,28,29]. Other modalities such as recall paradigm where the subject is asked to repeatedly recall emotional instances from their life and dyadic interaction where a facilitator helps in inducing the various emotions are also used by researchers [30,31]. Audio-visual elicitation using short film clips is found to elicit the target emotion better [32,33] compared to the other modalities. Hence, in this work emotions were induced by using short video clips.

### Pilot study

One of the major tasks in inducing emotions using short audio-visual clips is to identify video clips that would elicit the target emotions better. For this, around 20 video clips per emotional state were collected from various sources on the internet, and a pilot study was conducted. Fifteen volunteers in the mean age of 25 years participated in the pilot study to rate the emotions they experienced when watching the video clips. Sixty audio visual clips (ten for each emotion) with the highest rating were chosen for data collection. The emotional state ‘anger’ was excluded for further study because of poor rating, which points back to the local culture.

### Emotion induction protocol

**Figure 2:**
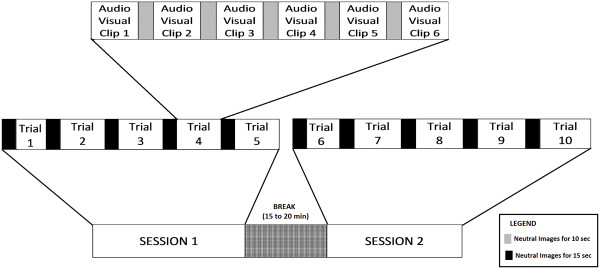
**Emotion elicitation protocol.** This is a schematic diagram that details the protocol used for data acquisition. Data was obtained in two sections with five trials each and a time gap allocated to relax between the sections.

The protocol used for data acquisition is as shown in Figure 2. There were two sessions with five trials in each section. Video clips pertaining to all the six emotional states (neutral, happiness, sadness, fear, surprise and disgust) were played in each trial in a predetermined random fashion. Care was taken not to play dimensionally opposite emotional video clips consecutively. Each of the emotional video clips lasted from 15 to 40 seconds, and was sandwiched between neutral images for 10 seconds. The neutral images between the video clips provided a small time gap for smooth transition between the emotional states. The entire protocol lasted for an hour with a break of 15 to 20 minutes between the two sessions. The participants were allowed to relax and refresh during the break.

### Participants

Sixty healthy volunteers, inclusive of thirty under graduate students from the university (18 to 25 years old), fifteen school children (9 to 16 years old) and fifteen adults (39 to 68 years old) participated in the data collection experiment. Each group had equal number of male and female participants. The participants of the pilot study did not take part in the data collection experiment.

The participants signed a consent form, after getting to know the purpose and procedure of the experiment. For children under the age of eighteen, consent was obtained from their parents or teacher. The experiment process complied with the recommendations of the AMA’s Declaration of Helsinki for human studies and the institutional policies.

### Procedure

In this work, ECG and EMG data were acquired simultaneously as the subjects watched the emotional video clips displayed on the screen using a self guided protocol. However, in this work we focus only on ECG signals. Power Lab data Acquisition System developed by AD Instruments, Australia was used to collect the emotional ECG data. Three electrodes were used; two active electrodes were placed on the hands (left and right) and one reference electrode on the left leg. The sampling frequency was set to 1000 Hz.

**Figure 3:**
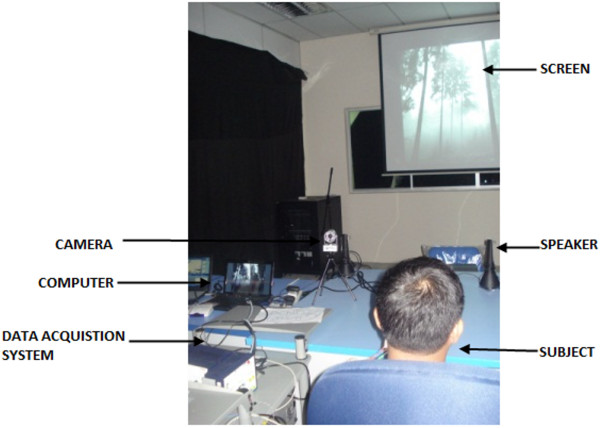
**Experiment setup for emotion assessment using audio-visual stimuli.** The controlled environment used for data collection along with the data collection equipments, laptops, screen and other details.

The subjects were requested to relax, minimize movement and concentrate on the audio-visual clips before starting the experiment. The set-up of the experiment is shown in Figure 3. The subjects watched the video clips on the LCD screen placed at a distance of seven meters in front of them. The video clips were played in the same order for all the subjects. After the experiment, they filled a self assessment questionnaire identifying the emotional state they experienced during the experiment. They also rated the intensity of their emotional state on a five point scale (1-very low to 5-very high). These ratings were then used to understand the intensity of the emotional state they experienced. However, despite the intensity levels, all the emotional data was taken into consideration and were randomized during processing.

### Data processing

**Figure 4:**
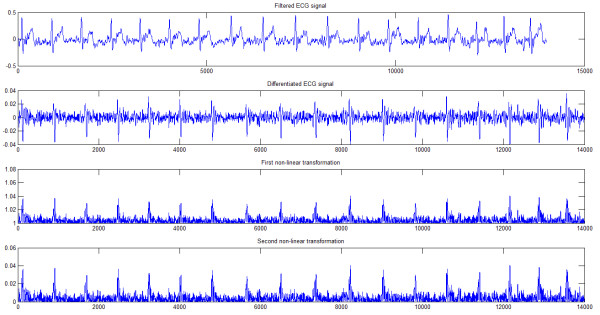
**QRS detection methodology.** The results different steps in deriving the QRS complex from the pre-processed ECG signals are illustrated.

The raw ECG data was split as per the emotional states and the noises that occur due to power line interference, muscle and movement artifacts were removed. Baseline wander that occurs at low frequency was removed by using the wavelet based algorithm proposed by Bunluechokchai et al., [34]. High frequency noises and power line interference were removed by using a 6th order Butterworth filter with a cut off frequency of 45 Hz. The reliability of the acquired signals was measured using the NN/RR ratio, where NN refers to the number of normal to normal beat intervals and RR refers to the total number of RR intervals in the ECG signal. Records with ratio less than 90% were excluded from further processing [17]. The QRS complex was derived by performing a non-linear transformations on the first derivative of the filtered ECG signal [35]. Figure 4 depicts the various stages in obtaining the QRS complex. The QRS peaks are distinctly seen after the second non-linear transformation. Hurst exponent and the proposed HOS features were computed from the QRS complex using two methods – Rescaled Range Statistics (RRS) and Finite Variance Scaling (FVS). It should be noted that the features were extracted from the QRS complex and not from HRV signals.

### Rescaled range statistics

This method analyzes the smoothness of a fractal time series based on the asymptotic pattern of the rescaled range of the process. First, the accumulated deviation of mean of time series over time is computed. The rescaled range R/S follows a power law relationship with time T as,

(1) $$R/S\sim T^{H}$$

R is the difference between the maximum and minimum deviation from the mean and S represents the standard deviation. Hurst, H is then derived as,

(2) $$H=\log\;\left(R/S\right)/\;\log\;\left(T\right)$$

where T is the length of sample data and R/S represents the corresponding value of rescaled range [20].

### Finite variance scaling

Finite variance scaling method is also known as standard deviation analysis and is based on the standard deviation D(t) of the variable x(t).

Considering the time series x(t) to be of length n, the standard deviation is computed as,

(3) $$D\left(t_{j}\right)={\left[\left\{\frac{\sum_{i=1}^{j}x^{2}\left(t_{i}\right)}{j}\right\}-{\left\{\frac{\sum_{i=1}^{j}x\left(t_{i}\right)}{j}\right\}}^{2}\right]}^{1/2}$$

for j = 1,2,….,n.

Eventually,

(4) $$D\left(t\right)\propto t^{H}$$

where H is the Hurst exponent. It is evaluated by finding the gradient of the best fitted log-log plot of D(t) and t [22].

### Proposed higher order statistics (HOS) based hurst features

HOS descriptors of order greater than two [36] retain finer information from the data and are appropriate for non-Gaussian and non-linear data [37,38]. Skewness and Kurtosis are normalized versions of third and fourth order cummulants respectively. Skewness measures the symmetry of a distribution around its mean and Kurtosis measures the relative heaviness of the tail of a distribution with respect to its normal distribution [24].

Let S(t) be the rescaled or finite variance scaled data defined by equations (1) and (4) respectively. Now generalizing and approximating the proportionality to equal we get,

(5) $$S\left(t\right)=t^{H}$$

Subtracting the mean μ_s_ and standard deviation σ_s_ of S(t) on both sides of the equation, cubing them and normalizing with length N, we get,

(6) $$\frac{1}{N}{\left[\frac{\left(S\left(t\right)-\mu_{s}\right)}{\sigma_{s}}\right]}^{3}=\frac{1}{N}{\left[\frac{\left(t^{H}-\mu_{s}\right)}{\sigma_{s}}\right]}^{3}$$

This equation can also be rephrased as,

Skewness,

(7) $$S_{\mathrm{skewness}}=\frac{{\left(t^{H}-\mu_{s}\right)}^{3}}{N\sigma_{s}^{3}}$$

Eventually,

(8) $$\left[\frac{N\sigma_{s}S_{\mathrm{skewness}}+\mu_{s}^{3}}{t^{3}-3\mu_{s}t^{2}-3\mu_{s}^{2}}\right]=t^{H}$$

Now, Skewness based Hurst,

(9) $$H_{\mathrm{skewness}}=\;\log\left[\frac{N\sigma_{s}S_{\mathrm{skewness}}+\mu_{s}^{3}}{t^{3}-3\mu_{s}t^{2}-3\mu_{s}^{2}}\right]/\;\log\left(t\right)$$

A relation for Kurtosis similar to equation 8 can be constructed in the same way but for order 4 as,

(10) NσsNS+kurtosis3−μs4t4+6t2μs−4μst3−μs2=tH

Now, Kurtosis based Hurst,

(11) $$H_{\mathrm{kurtosis}}=\log\left[\frac{N\sigma_{s}\left(NS_{\mathrm{kurtosis}}+3\right)-\mu_{s}^{4}}{t^{4}+6t^{2}\mu_{s}-4\mu_{s}\left(t^{3}-\mu_{s}^{2}\right)}\right]/\;\log\left(t\right)$$

Skewness based Hurst and Kurtosis based Hurst are non-linear, higher order features that can be easily computed.

### Classification of emotional states

Sixty subjects with six emotions and ten trials per emotion resulted in a total of 3600 samples. However, four trials of one subject had loose electrode contact because of which the data was ignored. Data from four kids and three young adults were also ignored because of unreliability captured using the NN/RR ratio. This resulted in a total of 3300 samples, which were processed. All the six features were extracted from these samples.

The performance of the different features as analyzed by four classifiers – Regression tree, naïve Bayes, K- Nearest Neighbour (KNN) and fuzzy KNN (FKNN). Regression tree classifier creates a decision tree for predicting the classes based on Gini’s diversity index whereas bayesian classifier is a probabilistic classifier based on Bayes theorem with strong independence assumptions. KNN and FKNN assigns a class based on the predominant class among the k nearest neighbors. The value of k was chosen to vary from six to fifteen as the number of classes used for classification here is six. Euclidean distance was used as the metric in KNN and FKNN allocates fuzzy class membership before making decisions.

In this work, random-cross validation was done to test the performance of the classifiers. The features derived from all the subjects were permutated and then categorized into 70% and 30% for all the six emotional states. Then the 70% features were used for training the classifier and 30% features were used for testing. The testing and training features belonged to random subjects and varied in each run of the program. However, they were mutually exclusive. Subject independent validation (also called leaving-one-person-out) was also performed for the RRS and FVS based combined analysis [39]. The features derived from 38 subjects were used for training the system and the other 16 subjects were used for testing adhering to the 70–30 rule. The classification accuracy is computed for the different emotional states as,

(12) $$\mathrm{\%Accurac}y_{\mathrm{Emotion}}=\frac{\mathrm{Number of correctly classified sample}s_{\mathrm{Emotion}}}{\mathrm{Total number of tested sample}s_{\mathrm{Emotion}}}\times 100$$

where *Emotion* refers to the six emotional states namely happiness, sadness, fear, surprise, disgust and neutral. The average accuracy was computed by taking the mean of the accuracies of all the six emotional states.

## Results

### Statistical data analysis

**Figure 5:**
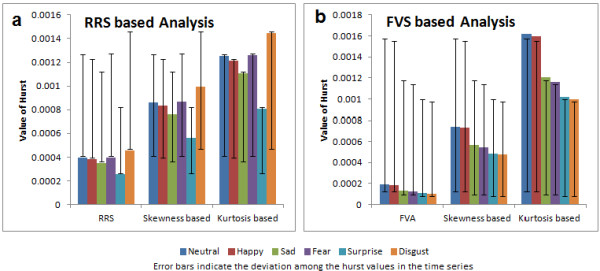
**Values of Hurst.** (**a**) RRS based analysis, (**b**) FVS based analysis. The values of hurst, skewness based hurst and kurtosis based hurst obtained in both the methods for all the six emotional states are plotted by means of a bar chart.

Hurst was computed using RRS and FVS methods for high (0.15 to 0.4 Hz), low (0.04-0.15 Hz) and very low frequency ranges (<0.04 Hz). These frequency ranges were chosen as they are widely used in emotion recognition algorithms using physiological signals [40]. Furthermore, researchers have also identified the impact of the QRS complex on low frequency oscillations which makes it important to analyze the low frequency range [41,42]. ANOVA indicated statistically significant (p < 0.001) changes among the six emotional states for Hurst computed in very low frequency range. The mean value of Hurst, in a very low frequency range for all the emotional states is as shown in Figure 5. We can observe that the value of Hurst ranges from 0.0002 to 0.0018 for all the features, which is similar to the work in [43] where the scaling exponent α is close to 0 for healthy heart beat data on a very low frequency range. Also in [44], the Hurst exponent of short term ECG series is close to 0 in the low frequency range during normalcy (pre- anesthetic stage).

**Figure 6:**
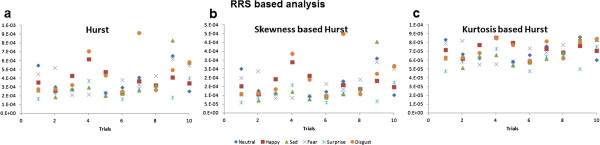
**RRS based Hurst analysis.** (**a**) hurst (**b**) skewness based hurst (**c**) kurtosis based hurst. The values of hurst exponent computed using the three RRS based methods for the six emotional states are plotted for the ten trials of subject 1.

**Figure 7:**
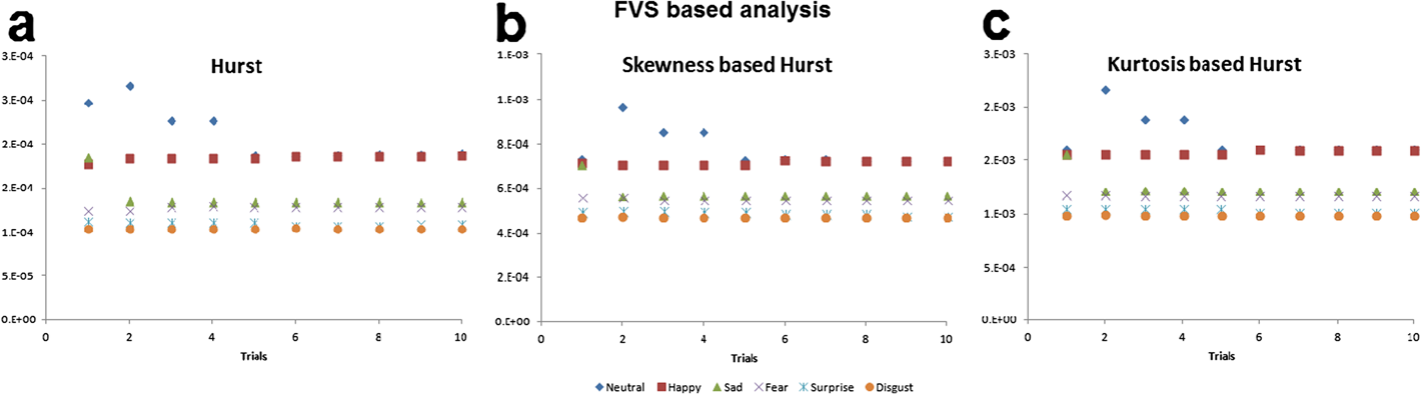
**FVS based Hurst analysis.** (**a**) hurst (**b**) skewness based hurst (**c**) kurtosis based hurst. The values of hurst exponent computed using the three FVS based methods for the six emotional states are plotted for the ten trials of subject 1.

We can observe from Figure 5 that the values of Hurst computed using RRS and FVS were very small in contrast to skewness based Hurst and kurtosis based Hurst which has larger values in both methods. Also, from Figure 5, significant differences cannot be perceived visually among all the emotional states. Happiness and neutral state seem to coincide in most of the cases whereas disgust and neutral state show disparity. The values of Hurst computed using RRS and FVS based methods for subject 1 are as shown in Figures 6 and 7 respectively. We can see that Hurst computed using RRS based method overlaps among the different emotional states. The range of variation is very large which leads to poor prediction of emotional states. In the case of FVS (Figure 7), a vague demarcation can be seen in the values of Hurst among the different emotional states. The emotions neutral and happiness overlap at many trials. Similar to disgust and surprise only a little variation can be seen among the emotional states sadness and fear. However, as the values of Hurst are not wide spread among all the emotional states, which may lead to better prediction.

The statistical significance of all the three features computed using both methods was studied using Analysis of Variance (ANOVA). The threshold was set to (p = 0.05) and all the three features for both RRS and FVS showed statistical significance (p < 0.001) indicating that the six emotional states have significant difference among them in the feature values. This also ensures the probability of achieving better classification accuracy.

The correlation among the different emotional states for the three features in both methods was studied using post hoc test of ANOVA. Least Significant Difference (LSD) was used to explore pair-wise comparisons of the six emotional states. In the case of RRS, the emotions sadness, surprise and disgust were significant with all the other emotional states (p < 0.001) for hurst and skewness based hurst. Kurtosis based hurst showed non-significance (p > 0.5) among the emotional states happiness, disgust and fear. In accordance with Figure 5, the emotional state neutral had non-significant correlations with happiness (p > 0.5) for all the features. Similarly, happiness and fear were not significant (p > 0.5). The pair wise combination of all other emotional states showed significance (p < 0.05) for all the features.

The features hurst and HOS based hurst computed using FVS showed significance (p < 0.001) in all the cases except the emotional states surprise and disgust (p > 0.3). The traditional hurst also lacked significance between the emotional states sadness and fear (p > 0.08). However, the pair wise comparisons of FVS based features were significant in almost all the emotional states.

### Classification results

The results of the three features extracted from the QRS complex using RRS based method is tabulated in Table 1. We can observe that the average accuracy of 82.88% is the highest for Skewness based Hurst classified using FKNN classifier. In general, the maximum average accuracy of all the features varied from 60.44% to 82.88% depending on the classifier used. Skewness based hurst showed better results with a maximum average accuracy of 82.88% compared to kurtosis based hurst which has a maximum average accuracy of 60.44%. However all the features computed by RRS did not suit the Bayesian classifier which shows very less results for all the features and all the emotional states, more specifically for the emotion disgust which lies in the fourth quadrant of the valance-arousal plot (Figure 1).

**Table 1:** RRS based analysis for emotion classification

**Feature**	**Classifier**	**Neutral**	**Happy**	**Sad**	**Fear**	**Surprise**	**Disgust**	**Average**
		**(%)**	**(%)**	**(%)**	**(%)**	**(%)**	**(%)**	**(%)**
Hurst	Regression Tree	62.91	76.00	67.78	71.81	73.83	59.06	68.56
	Bayesian Classifier	76.82	61.33	19.46	10.07	39.00	13.42	36.78
	KNN (K = 7)	71.52	74.00	73.83	73.15	71.81	57.04	70.23
	FKNN (K = 8)	61.59	72.00	65.10	73.83	73.15	64.43	68.35
Hurst_skewness_	Regression Tree	64.24	62.00	66.44	64.43	68.46	59.73	64.22
	Bayesian Classifier	81.46	73.33	14.77	11.41	28.19	11.41	36.76
	KNN (K = 7)	68.21	64.67	63.09	68.46	67.79	61.75	65.66
	FKNN (K = 10)	63.57	60.00	60.40	66.44	7.79	59.06	82.88
Hurst_kurtosis_	Regression Tree	62.91	56.00	60.40	65.10	71.14	42.95	59.75
	Bayesian Classifier	29.80	65.33	49.66	12.75	34.89	11.41	33.98
	KNN (K = 10)	56.29	56.67	59.06	67.79	75.17	47.65	60.44
	FKNN (K = 10)	54.97	56.00	59.06	65.10	71.14	47.65	58.98

Table 2 shows the classification results of FVS based analysis. The accuracy of hurst is similar to the RRS based method with the maximum average accuracy of 65.33% using the FKNN classifier. Here, kurtosis based hurst performs better with a maximum average accuracy of 81.72% compared to hurst and skewness based Hurst for which the values are 65.33% and 74.66% respectively. HOS features show improved performance compared to the RRS based method.

**Table 2:** FVS based analysis for emotion classification

**Feature**	**Classifier**	**Neutral**	**Happy**	**Sad**	**Fear**	**Surpris**	**Disgus**	**Average**
		**(%)**	**(%)**	**(%)**	**(%)**	**e (%)**	**t (%)**	**(%)**
Hurst	Regression tree	54.72	61.90	67.12	65.07	60.96	76.03	64.30
	Bayesian classifier	41.22	48.30	46.58	78.10	36.99	92.47	57.27
	KNN (K = 6)	58.78	62.58	64.38	65.06	64.38	82.19	66.23
	FKNN (K = 11)	55.40	59.18	69.17	63.01	66.43	78.76	65.33
Hurst_skewness_	Regression tree	61.49	64.63	78.08	77.39	73.97	82.19	72.95
	Bayesian classifier	29.05	79.59	53.42	71.91	73.97	43.84	58.63
	KNN (K = 8)	59.45	62.58	69.86	85.61	74.65	79.45	71.93
	FKNN (K = 8)	66.21	61.90	76.71	80.13	77.39	85.61	74.66
Hurst_kurtosis_	Regression tree	68.91	64.62	84.24	91.09	84.25	90.41	80.59
	Bayesian classifier	44.59	70.74	61.64	79.45	36.30	100	65.45
	KNN (K = 10)	67.56	67.34	80.13	91.09	83.06	95.20	80.81
	FKNN (K = 10)	72.97	65.30	85.61	90.41	86.30	89.72	81.72

Skewness based Hurst and Kurtosis based Hurst, being features extracted using higher order statistical methods retain finer emotional information in the signal. Hence, they show better performance compared to the normal Hurst which is very much obvious in the FVS method. Also, the FVS based features coped up with all the classifiers.

The results obtained by combining all the features of RRS and FVS based methods using random validation are shown in Table 3 and Table 4 respectively. We can see improved results in the case of FVS compared to RRS. The combined analysis of RRS performed similar to the individual analysis having a maximum average accuracy of only 65.45%. The FVS based combined analysis shows a maximum classification accuracy of 92.87% using the FKNN classifier. In general, the accuracy of the individual emotional states is also higher in the FVS combined analysis compared all other analysis.

**Table 3:** RRS based combined analysis for emotion classification (random validation)

**Classifier**	**Neutral**	**Happy**	**Sad**	**Fear**	**Surprise**	**Disgust**	**Average**
	**(%)**	**(%)**	**(%)**	**(%)**	**(%)**	**(%)**	**(%)**
Regression tree	59.60	58.00	69.12	69.13	67.78	61.07	64.12
Bayesian classifier	39.07	64.67	71.14	18.79	36.91	10.74	40.22
KNN (K = 11)	66.89	56.67	75.83	68.46	67.79	57.05	65.45
FKNN (K = 12	65.56	54.00	70.47	70.47	68.45	55.70	64.11

**Table 4:** FVS based combined analysis for emotion classification (random validation)

**Classifier**	**Neutral**	**Happy**	**Sad**	**Fear**	**Surprise**	**Disgust**	**Average**
	**(%)**	**(%)**	**(%)**	**(%)**	**(%)**	**(%)**	**(%)**
Regression tree	72.29	73.46	91.78	88.36	91.10	97.26	85.71
Bayesian classifier	26.35	83.67	57.36	71.91	61.64	100	65.82
KNN (K = 6)	63.51	83.67	89.04	95.21	95.21	100	87.72
FKNN (K = 6)	78.38	91.16	95.21	95.21	98.63	98.63	92.87

Also, from Table 4, we can also observe that the emotional state disgust belonging to negative valance (quadrants 4 of Figure 1) was correctly classified for most of the test samples. The accuracy is less for the emotional states neutral and happiness.

**Figure 8:**
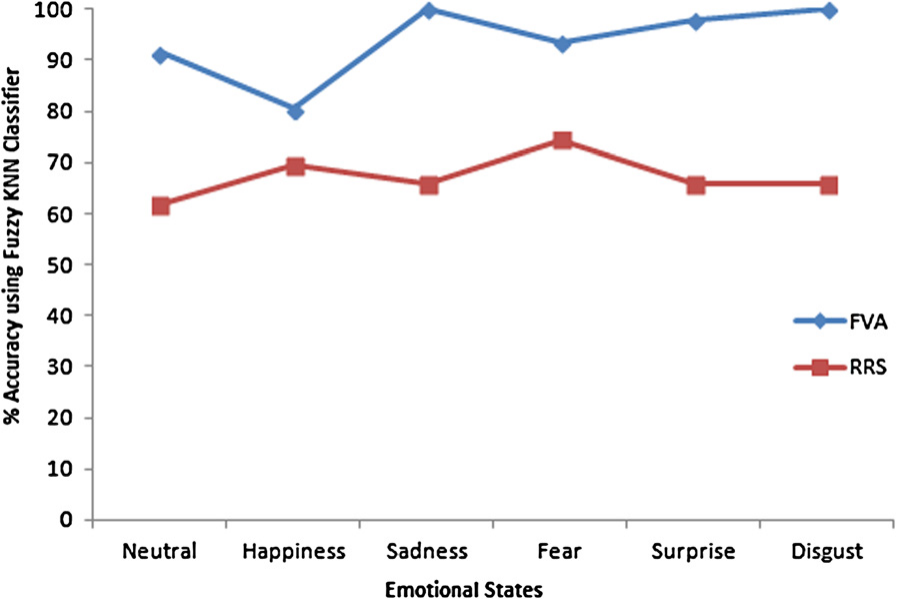
**Comparison of RRS and FVS based analysis.** This graph plots the highest accuracy for all the emotional states obtained in both the methods. FVS based method performs much better compared to RRS based method.

The comparison chart of the RRS and FVS based combined analysis is illustrated in Figure 8. The traces show the accuracy values of all the emotional states classified using FKNN classifiers which are from the last rows of Table 3 and Table 4 for the RRS and FVS based analysis respectively. The figure shows that FVS based analysis works better for all the emotional states with an overall higher performance. The performance of the emotion disgust is contrasting with the least performance in the RRS combined analysis (55.70%) and the best performance in FVS based analysis (98.63%). These results indicate that the features obtained by combining FVS and HOS are able to retain more emotional information without varying the value of Hurst.

Tables 5 and 6 show the performance of the subject independent classification for the RRS based combined analysis and FVS based combined analysis. We can observe that the RRS based combined analysis performs almost similar with a maximum average accuracy of 65.45% and 66.71% for both random validation and subject independent validation respectively. However, the FVS based combined analysis shows differing results. The maximum average accuracy of the random validation method is 92.87% in contrast to the maximum average accuracy of the subject independent validation which is 76.45%. In both cases, the FVS based combined analysis performs better compared to RRS based methods. However, the performance of the emotion recognition system needs to be further improved in subject independent analysis.

**Table 5:** RRS based combined analysis for emotion classification (subject independent validation)

**Classifier**	**Neutral**	**Happy**	**Sad**	**Fear**	**Surprise**	**Disgust**	**Average**
	**(%)**	**(%)**	**(%)**	**(%)**	**(%)**	**(%)**	**(%)**
Regression tree	60.37	67.50	66.24	59.12	72.61	63.06	64.82
Bayesian classifier	69.81	60.00	29.24	30.19	29.94	9.55	38.13
KNN (K = 11)	63.52	67.50	64.33	66.04	73.89	64.97	66.71
FKNN (K = 12	60.37	61.88	70.06	62.89	70.06	64.33	64.93

**Table 6:** FVS based combined analysis for emotion classification (subject independent validation)

**Classifier**	**Neutral**	**Happy**	**Sad**	**Fear**	**Surprise**	**Disgust**	**Average**
	**(%)**	**(%)**	**(%)**	**(%)**	**(%)**	**(%)**	**(%)**
Regression Tree	50.02	52.66	78.91	79.86	95.91	81.75	73.69
Bayesian Classifier	17.44	90.00	27.89	72.48	37.41	90.54	53.96
KNN (K = 11)	44.29	64.66	65.98	90.60	93.87	94.59	75.67
FKNN (K = 12	44.97	65.33	65.98	91.95	94.55	93.92	76.45

## Discussion

### Necessity of non-linear analysis

A number of research works have been done to recognize emotional states using one or more physiological signals. Many researchers have worked on identifying two, three or four emotional states mainly dealing with the valance scale or the four quadrants of the valance-arousal diagram (Figure 1). So far, a maximum mean classification rate of 95% and 70% has been achieved on recognizing four emotions (joy, anger, sadness, pleasure) in user dependent and user independent approach, respectively [2]. Similarly, 86% and 45% accuracy has been obtained for detecting two (joy, sadness) and six (amusement, contentment, disgust, fear, neutrality, sadness) emotions respectively in a user independent approach [15,45].

The accuracy of the various methods differs because of a number of factors such as: type and number of physiological signals used, placement of electrodes, number of subjects, age range of subjects, attention and orientation of subjects, number of emotions considered, type of emotion induction, familiarity of protocol and type of signal analysis. So far, there is no standard set up in recognizing emotions from physiological signals [46].

Furthermore, most of these works use the statistical features proposed by Picard and linear or non-linear classifiers. Non-linear analysis using empirical mode decomposition for classifying four emotions has resulted in an accuracy of 76% for user-independent approach [3]. Valenza et al., in their work proved nonlinear methods to identify four different valance levels with an accuracy higher than 80%. The neutral state was captured with 96.78% and valance 3 with 100% accuracy [47] Nonlinear dynamics has also been found powerful in estimating the instantaneous heart beat dynamics involved in autonomic control [48-51]. All these works indicate that non-linear methods can be used to extract emotional features from physiological signals in a better way. This work uses the non-linear feature Hurst which provides a higher accuracy of 92.87% and 76.45% in random and subject independent validations respectively compared to previous research works on classifying six emotions in a user independent way.

The tables also indicate that the higher results were obtained for the FKNN classifier which works on membership functions assigned to the different test features. KNN and regression tree classifiers also performed better with almost similar results. The Bayesian classifier did not perform well in almost all the classes indicating that the independent probabilistic assumptions made by the classifier did not suit the classification of emotional states.

By trying to utilize the advantage of HOS in retaining minute information of the signal, we can observe that a maximum average accuracy of 92.87% is obtained for classifying six emotional states in using random validation. The classifiers also performed better than the existing methods with a maximum average accuracy of 76.45% when validated in a subject independent way. Though the value of Hurst was similar to the traditionally obtained results, HOS based methods were able to capture emotional variation in the ECG signals compared to the traditional analysis. Furthermore ECG signals being complex, non-linear and non-stationary, non-linear analysis would pave a better way to capture minute and infinitesimal changes that are prone to occur during emotional changes.

### Real time emotion recognition

Emotions that are expressed in a controlled laboratory environment may not be similar to how they are expressed in the natural world [4]. Also the intensity of emotions and changes in physiological signals associated with emotions varies from person to person. The age and gender of a person also play a role in the emotions experienced [29]. K. H. Kim et al., worked on children with ages ranging from five to eight years to classify three and four emotional states [28]. Furthermore emotions also depend on the culture. Our pilot study showed that most of the subjects hardly felt angry, which is common in this region.

Emotions are transient and they occur only for a small instant of time though they influence the way we process information through attentional or perceptual biases [52]. Though the emotion felt is not really visible, the consequences or action lead by the emotion is visible.

A real time system should be able to tackle the subjective dependency of emotions and also track the transient changes in emotional levels of the physiological signal. This would require a more generalized and authentic system with robust and reliable signal processing algorithms that could be fast enough in deciphering the short-lived emotional state of the subject. The robust algorithm should also be simple enough to be computed in real time.

Furthermore, analysis needs to be done with data from different age groups, ethnicities and backgrounds. Experiments need to be carried out by using the different modalities of emotion recognition and the variations among the modalities need to be studied. The intensity of emotion experienced by the subject should be considered when analyzing the data. An extensive data collection considering all the issues and developing a robust algorithm would help in developing a reliable real-time system.

### Limitations of the study

Emotions are also dependant on a number of variables such as room temperature, time of day, circadian rhythm, position, activity level of the subject before recording, medication, hormone levels, verbalization and breathing condition. Though much care was taken to eliminate these issues by allowing the subject to choose their own free time for participating in the experiment and relax by means of some breathing exercise before the start of the experiment, more care should be taken to consider these differences as well when developing a real time emotion recognition system. The impact of these differences on the emotional state of the person also needs to be studied.

## Conclusions

This study indicates that ECG signals are reliable in identifying the true emotional state of a person. The design of data acquisition protocol for eliciting the six emotional states (happiness, sadness, fear, surprise, disgust and neutral) and the data acquisition methodology are explained in detail. Two new methods to compute the non-linear feature Hurst by combining the normalized HOS parameters and the traditional Hurst computation methods are proposed. The performances of the different features were analyzed using four classifiers namely regression tree, naive bayes, k-nearest neighbor (KNN) and fuzzy k-nearest neighbor (FKNN). The Hurst computed using FVS and HOS yields better results of 92.87% and 76.45% for random and subject independent validation respectively using FKNN classifier. Computing Hurst by combining HOS with traditional methods retains the advantage of both HOS and non linear method, enabling to identify the minute emotional changes that occur in any healthy ECG data. This algorithm can be studied further by trying to combine HOS with other non linear features. Also, an extensive data analysis is required towards the development of a real trime and robust emotion recognition system.

## Abbreviations

ANS: Autonomous nervous system; ECG: Electrocardiogram; HRV: Heart rate variability; EEG: Electroencephalogram; EMG: Electromyogram; GSR: Galvanic skin response; BVP: Blood volume pressure; ST: Skin Temperature; ANOVA: Analysis of variance; FVS: Finite variance scaling; RRS: Rescaled range statistics; HOS: Higher order statistics; APEN: Approximate entropy; LLE: Largest lyapunov exponent; CD: Correlation dimension; H: Hurst exponent; WT: Wavelet transform; EMD: Empirical mode decomposition; KNN: K nearest neighbor; FKNN: Fuzzy K nearest neighbor; AMA: American medical association.

## Competing interests

The authors declare that they have no competing interests.

## Authors’ contributions

JS carried out the data acquisition and analysis, participated in the sequence alignment and drafted the manuscript. MM conceived of the study, and participated in its design and coordination and helped to draft the manuscript . KW and SY participated in the design of the study and data analysis methods. All authors read and approved the final manuscript.
